# Functional roles and novel tools for improving‐oxidative stability of polyunsaturated fatty acids: A comprehensive review

**DOI:** 10.1002/fsn3.3272

**Published:** 2023-02-28

**Authors:** Fakhar Islam, Ali Imran, Farhana Nosheen, Maleeha Fatima, Muhammad Umair Arshad, Muhammad Afzaal, Nosheen Ijaz, Rabia Noreen, Shilpa Mehta, Sunanda Biswas, Izza Faiz Ul Rasool, Muhammad Arslan Aslam, Ifrah Usman, Syeda Mahvish Zahra, Narimane Segueni, Yuosra Amer Ali

**Affiliations:** ^1^ Department of Food Science Government College University Faisalabad Faisalabad Pakistan; ^2^ Department of Home Economics Government College University Faisalabad Faisalabad Pakistan; ^3^ Department of Electrical and Electronic Engineering Auckland University of Technology Auckland New Zealand; ^4^ Department of Food and Nutrition Acharya Prafulla Chandra College Kolkata India; ^5^ Department of Environmental Design, Health and Nutritional Sciences Allama Iqbal Open University Islamabad Pakistan; ^6^ Institute of Food Science and Nutrition University of Sargodha Sargodha Pakistan; ^7^ Faculty of Medicine University Salah Boubnider Constantine 3 Constantine Algeria; ^8^ Department of Food Sciences, College of Agriculture and Forestry University of Mosul Mosul Iraq

**Keywords:** health benefits, nanoencapsulation, oxidative stability, polyunsaturated fatty acids

## Abstract

Polyunsaturated fatty acids may be derived from a variety of sources and could be incorporated into a balanced diet. They protect against a wide range of illnesses, including cancer osteoarthritis and autoimmune problems. The PUFAs, ω‐6, and ω‐3 fatty acids, which are found in both the marine and terrestrial environments, are given special attention. The primary goal is to evaluate the significant research papers in relation to the human health risks and benefits of ω‐6 and ω‐3 fatty acid dietary resources. This review article highlights the types of fatty acids, factors affecting the stability of polyunsaturated fatty acids, methods used for the mitigation of oxidative stability, health benefits of polyunsaturated fatty acids, and future perspectives in detail.

## Graphical abstract

This review article highlights the types of fatty acids, factors affecting the stability of polyunsaturated fatty acids, methods used for the mitigation of oxidative stability, and the health benefits of polyunsaturated fatty acids.

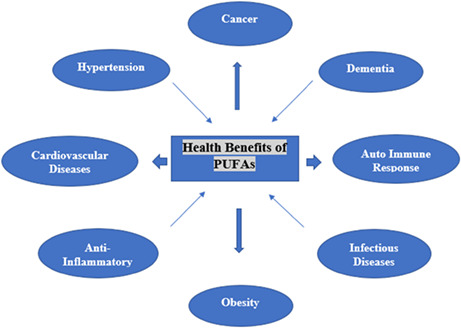

## 1 INTRODUCTION

Polyunsaturated fatty acids (PUFAs) are essential according to the health point of view due to the occurrence of more than one unsaturated bond in their molecules. Fatty acids are further divided into α‐linolenic acid (ALA) and linoleic acid (LA), as well as fatty acids having long chain, including docosahexaenoic acid (DHA), arachidonic acid (AA), and eicosapentaenoic acid (EPA), which is divided further into two classes such as DHA, ALA, EPA, and ω‐3 along with ω‐6, such as AA and LA are very popular, common, and most studied fatty acids (Kus‐Yamashita et al., 2016). According to the last few decades, PUFAs express advantages for public health through having a part in the maintenance of inflammation, brain function, and cholesterol metabolism (Palacios et al., 2022). Vegetable oils that are extracted from different plant seeds are an abundant source of MUFAs and PUFAs (Pattnaik & Mishra, 2022). Despite early interest in the health benefits of polyunsaturated fatty acids (PUFAs), the data connecting PUFAs to cardiovascular disorders are still highly contested and ambiguous (CVDs). Using Mendelian randomization, we examined the impact of plasma concentrations of ω‐3 PUFA (docosahexaenoic acid [DHA] and total ω‐3) and ω‐6 PUFA (linoleic acid and total ω‐6) on the risk of CVDs, Borges et al. (2022). Sunflower oil, fish oil, and groundnut oil form rancid odors owing to oxidative degradation because of the elevated levels of MUFA and PUFA (Peter et al., 2022). WHO reported that about 41 million deaths in 2018 were recorded just because of noncommunicable diseases. In developing the threat of noncommunicable chronic disorders, both inflammation and oxidative stress are major contributors. PUFAs influence both the antioxidant and inflammatory signaling pathways (Djuricic & Calder, 2021). PUFAs also work as antioxidants through antioxidant signaling pathway regulation and inflammatory cycle modulation (Calder, 2020). Lipid oxidation is completed in three stages named as initiation, reproduction, and termination. Fatty acid radicals such as LOO˙, LO˙, and L˙ are the most significant free radicals in the process of oxidation (Li et al., 2022). Tendency of polyunsaturated fatty acids (PUFAs) for going through oxidation plays a vital role in the integrity of biological membrane and lipid‐containing foods (Dessì et al., 2002). ω‐3 (PUFAs) like EPA and DHA are popular and beneficial for providing protection against different types of metabolic disorders (Saini et al., 2021). ω‐3 PUFAs have the potential to promote health by decreasing risks of inflammation, cardiovascular disorder, and cancer. Previous literature shows that the proportion of lipid oxidation is increased by increasing the concentration of unsaturation and that is why ω‐3 PUFAs are extremely susceptible for oxidation due to the presence of double bonds (Zhang et al., 2020). Likewise, PUFAs are most susceptible for oxidation due to their unsaturated bond having nature that generates different metabolites along with reactive oxygen species. Both levels of oxidation and metabolites may affect the effectiveness of PUFAs in a positive beneficial or negative way. As the presence of multiple unsaturated bonds, PUFA is more susceptible for oxidation process and then this oxidation process is further characterized into two different types named as nonenzymatic oxidation and enzymatic oxidation. Nonenzymatic oxidation then further branched into autoxidation (mediated by free radicals) and photo‐oxidation (facilitated by singlet oxygen or ultraviolet) (Tao, 2015). Edible oils are going through deterioration processes during their storage, cooking, and handling owing to lipid oxidation. The oxidation mechanism is responsible for the loss of any oil quality, decreasing the nutritional content of the oil, or producing unwanted off‐flavors, making oils containing food less appealing to customers. Moreover, lipid oxidation generally results in the formation of hazardous by‐products including reactive carbonyl compounds (RCCs), which might result in the formation of end products of novel lipid peroxidation that potentially destroys the health of people. The presence of antioxidants, the usage of oxygen, and the temperature all have an impact on lipid oxidation. Interaction of oils with oxygen may be higher for chain reactions that progress faster with high temperatures and increased unsaturation degree of lipids (Fadda et al., 2022). This review article highlights the types of fatty acids, factors affecting the stability of polyunsaturated fatty acids, methods used for the mitigation of oxidative stability, health benefits of polyunsaturated fatty acids, and future perspectives in detail.

## 2 POLYUNSATURATED FATTY ACIDS

**FIGURE 1:**
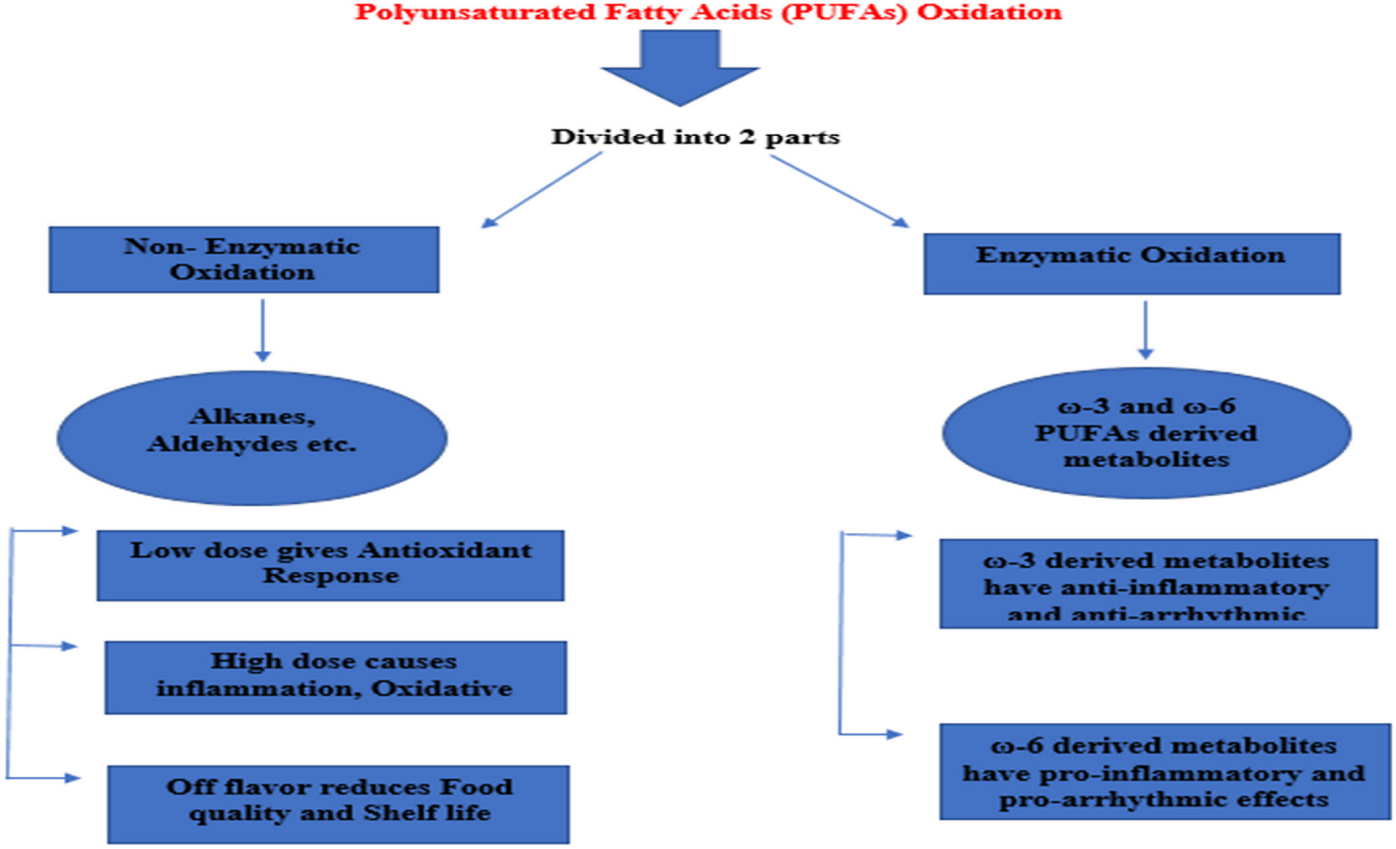
Flow sheet depicts the PUFAs oxidation.

Fatty acids are fat‐soluble portions either from plant or animal side that is the main integral of lipids. FAs are classified into three categories such as (1) saturated FAs (no double bonds), (2) MUFAs (single double bond), and (3) PUFAs (≥2 double bonds) (Saini & Keum, 2018). Due to the sequence of double bonds, unsaturated FAs are divided further into mono‐ and polyunsaturated fatty acids. As primary building blocks of cell membranes, PUFAs are also recognized as essential nutrients for treating autoimmune responses, chronic diseases, and nonalcoholic fatty liver (Lee et al., 2016). The PUFAs ‐3 and ‐6 are also named as essential fatty acids since neither would be produced by body and then obtained from food Figure 1.

Essential fatty acids like LA and ALA are vital PUFAs (Calder, 2017). Eicosanoids and docosanoids both are pro‐ and anti‐inflammatory lipid mediators, as well as precursors of PUFAs. Inflammatory and immunological responses are mediated by PUFA‐derived prostaglandins, prostacyclins, and leukotrienes. The formation of anti‐inflammatory eicosanoids uses EPA as a substrate (Kosmalski et al., 2022). The docosanoids, protectins, and resolvins, which have anti‐inflammatory and immunomodulatory properties, are precursors of DHA. Both PUFAs ω‐6 and ω‐3 are regulated as essential in avoidance of various undesired systemic responses, such as autoimmune response, due to their biological activities. Additionally, PUFAs play a vital role in chronic illnesses such as diabetes mellitus, cancer, and cardiovascular system (Czumaj & Śledziński, 2020). Consuming the plant seeds like fish, flax, and perilla seeds and chia oil helps in satisfying the body's need for ω‐3 fatty acids. Additionally, cereal products that are the top source of ω‐3 fatty acids. EPA, α‐linolenic acid (ALA), and docosahexaenoic acid (DHA) are further sources of ω‐3 fatty acids while LA (linoleic acid) and ARA (arachidonic acid) are crucial ω‐6 fatty acids. Vegetable oils like sunflower and soybean oils are main sources of ω‐6 fatty acids and have ω‐3 fatty acids in less amount (Kapoor et al., 2021). ALA produces EPA and DHA, which are the precursors of eicosanoids with anti‐inflammatory, antithrombotic, and antiarrhythmic effects through the inefficient enzymatic activity of desaturation (Simopoulos, 2006). Patients with atherosclerosis, obesity, dyslipidemia, metabolic disorder, hypertension, diabetes mellitus, infectious diseases, neurological/neuropsychiatric disturbances, and ocular diseases may get benefit from the consumption of dietary supplemented with ω‐3 fatty acids (Yashodhara et al., 2009). Mostly, cardiovascular disease liked mortality rates are very high in Western culture because they consume a high‐fat diet. Different studies had conducted for testing the effects of ω‐3 PUFAs on different cardiovascular disorders namely rhythm disorders, congenital heart disease, stroke, atrial fibrillation, myocardial infarction, coronary heart disease, subclinical atherosclerosis, sudden cardiac death, heart failure, peripheral arterial disease, and valvular disease (Writing Group Members et al., 2016). Different epidemiological researches showed that the lower intake level of ω‐3 PUFA is related to higher chances of cognitive failure or dementia particularly for Alzheimer's disease (Cole et al., 2009), Figure 2.

**FIGURE 2:**
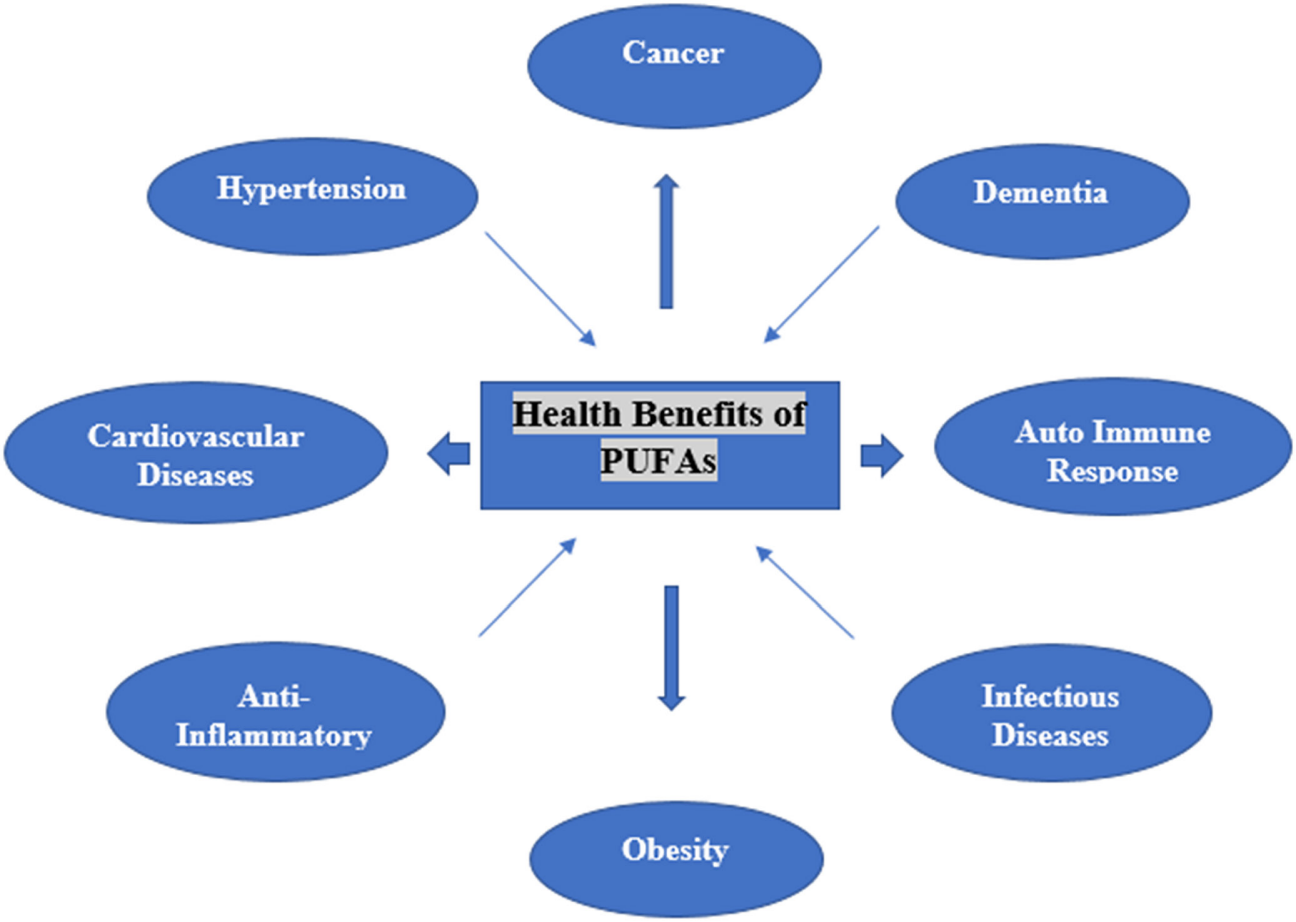
Depicts the health benefits of PUFAs.

## 3 FACTORS AFFECTING THE STABILITY OF POLYUNSATURATED FATTY ACIDS

### 3.1 Physical factors

Due to their straightforward structure, conventional emulsions are simple to prepare. Their ability to resist oxidation is greatly influenced by the emulsifiers employed. Due to the single layer of emulsifier in these emulsions, they are typically susceptible to environmental factors during storage, including pH, ion strength, and temperature. These elements might weaken emulsions physically, which would weaken PUFAs' chemical stability in the mixtures. Bilayer emulsions' thicker interfacial layer serves as a physical blockade, which separates PUFAs from pro‐oxidants in the phase of aqueous, which helps to reduce the oxidation of PUFA (Berton‐Carabin et al., 2015). The dried emulsions into powders and the physical blockade of a thick interface coating, in addition to dispersion systems, help to lower the oxidation rate of the microcapsules. Hydrolysates of whey and pectin protein stabilized bilayer emulsions, in which hydroperoxide concentration decreased by about 60% compared with the single layer microcapsules subsequently 12 weeks, were used to create bilayer microcapsules by spray‐drying (Tamm et al., 2016). When stored at various temperatures, the spray‐dried bilayer microcapsules from chitosan‐/lecithin‐stabilized emulsions had a greater antioxidant influence than the monolayer microcapsules. It is usual practice to make mixtures by means of three sheets of emulsifiers in an exertion further to increase PUFA oxidation resistance. Tween 20, pectin, and chitosan stabilized trilayer fish oil emulsions were produced by Jo et al. (2015). The findings showed that the number of interfacial layers had an influence on the oxidation steadfastness of fish oil in emulsions. Genipin is a naturally occurring connecting agent that interacts by means of proteins' main amine groups (like arginine and lysine) to establish covalent connections both inside and between molecules (Johnston et al., 2015). Emulsifiers can be modified by genipins to increase their physicochemical stability. Menhaden oil's improved oxidative stability was seen for emulsions stabilized with WPI‐epigallocatechin gallate and WPI following interfacial cross‐connecting by genipin (Fan et al., 2018). A thick and dense interfacial layer, which prevents the oxidation of lipid by delaying the penetration of pro‐oxidants and free radicals, may be produced when interfacial proteins are cross‐linked (Fan et al., 2018). An efficient method for altering the interfacial layer and enhancing PUFAs' resistance to oxidation is high‐pressure processing (Lee et al., 2009). After 10 days at 50°C, lipid hydroperoxide levels in treated, 120 MPa microfluidization emulsions were almost three times higher than those in untreated, gliadin particle‐stabilized emulsions. When a protein's disulfide bond unravels under high‐pressure, intermolecular disulfide connections are created. This irreversible structural alteration affects both adsorbed and free proteins, resulting in the formation of a stiffer interface layer that can effectively prevent the oxidation of PUFAs (Liu et al., 2016). There are additional chances for changing the interfacial structure because of interactions between biopolymers. Hydrogen bonds, electrostatic interactions, and hydrophobic interactions all play role in the physical complexation of biopolymers. These noncovalent complexes can be produced either during or after the manufacture of the emulsion and do aid in preventing the oxidation of PUFAs in emulsions (Huang et al., 2017). The process used to manufacture the former, electrostatic deposition, is mostly employed to create multilayer emulsions. Ability of proteins to fight free radicals is good and can even get better following fibrillation. Food proteins are always heated for several hours at a low pH and high temperature (usually pH 2, 80–90°C, for 5–24 h). Protein monomers unfurl, hydrolyze, and aggregate into amyloid fibrils upon heating, triggering a cascade of intricate physical and chemical processes. It is intriguing to consider how fibrillation affects emulsion stability since structural alterations may cause proteins' functional activity to be amplified. According to the studies, whey protein fibrils outperformed WPI in terms of reducing power, 2,2‐Di(4‐tert‐octylphenyl)‐1‐picrylhydrazyl (DPPH) radical scavenging activity, and 2,2′‐Azino‐bis (3‐ethylbenzothiazoline‐6‐sulfonate) (ABTS) free radical‐scavenging actions (Feng et al., 2018; Mohammadian & Madadlou, 2016). Natural antioxidant plant essential oils have recently been found to be able to change the characteristics of emulsions containing ω‐3 PUFAs while also having a potent capacity to neutralize free radicals. The collaboration between surfactants and other essential oils may thicken the interface layer, improving stability against oxidation. According to Mora‐Gutierrez et al. (2018), a DHA‐rich goat milk emulsion was supplemented with seed oil from hibiscus, which contains alpha‐tocopherol (21.4%), gamma‐tocopherol (78.2%), and δ‐tocopherol (0.4%).

### 3.2 Chemical factors

Various methods have been established to reduce PUFA oxidation, most notably the introduction of antioxidants, microencapsulation, and formulation of emulsion (Miyashita et al., 2018). Viscosity, pH, and aqueous phase ionic potential are additional issues, which affect the PUFA emulsions' oxidative stability. The assembly of the oil/water interface, from where PUFA oxidation started, was related to influence both oil phase and the aqueous phase on the safety of PUFA. The most significant elements influencing the pace at which PUFAs oxidize are thought to be the charge, thickness, and configuration of the interface (Cui et al., 2018; Szumałaa & Wysocka, 2018).

The antioxidation capacity of proteins varies owing to variable amino acid arrangements, its composition, and 3‐D layouts. Roughly proteins could also suppress lipid oxidation by free radical scavenging (Islam et al., 2023). When used as emulsifiers, whey protein, soy protein, egg protein, and casein, for instance, possibly increase PUFA oxidation stability. When associated with the casein‐stabilized emulsion at pH 7.0, the emulsion with whey protein stabilization demonstrated a reduced rate of PUFA oxidation. It was hypothesized that this occurred because the other protein carboxylate groups had a lower affinity for pro‐oxidants like iron than did the phosphate groups supported by the molecules of casein, which caused pro‐oxidants to aggregate at the interface and speed up the oxidation. The oxidative stability of PUFAs can be enhanced by polysaccharides that include both polar and nonpolar groups adhering to exteriors of oil droplets. For instance, adding pectin to Brij 35‐equilibrated emulsions successfully prevented menhaden oil from oxidizing. It happens because the pectin's capacity to chelate metals reduces the amount of oxidant substances in the continuous phase (Celus et al., 2018). When compared to traditional emulsions with a single interfacial layer, PUFAs in bilayer emulsions exhibit improved oxidation stability. Oil droplets that have been emulsified and are trapped inside of a gel matrix makeup gelled emulsions. They are often made by either directly mixing thickeners into the main emulsions or encouraging the emulsions' biopolymers (like polysaccharides and proteins) to cross‐link. The use of gelled emulsions by way of food‐grade delivery vehicles for PUFAs is the subject of current study. Studies have shown that PUFAs may be effectively protected against oxidation by being added to congealed emulsions (Farjami & Madadlou, 2017; Lu et al., 2019). The term “Pickering emulsions” describes emulsions that are stabilized through solid particles rather than surfactants. Those particles of solid permanently attach to droplet surfaces, lower surface tension, and generate more physically stable emulsions. Pickering emulsions are superior to ordinary emulsions in protecting PUFAs against chemical deterioration (Kargar et al., 2011). By using only solid particles as stabilizers, Pickering emulsion prevents droplets from coalescing by accumulating at the boundary between two immiscible liquids (sometimes referred to as the oil and water phase). Along with the anticipated outcomes of these methods on the emulsions, current methodologies and techniques for the examination of the development and characteristics of the pickering emulsion were described. A modeling approach application has also been developed for the accurate characterization of pickering emulsions (Low et al., 2020). A reasonably common natural antioxidant is polyphenol. The quantity and configuration of benzyl groups and phenolic hydroxyl groups determine its antioxidant activity. In an emulsion system, polyphenols could be adsorbed because of the oil–droplet junction hydrophobic interaction since a considerable amount of amphiphilicity exists in polyphenol itself. This prevents lipid oxidation at the oil–water junction. Additionally, the interface between interfacial surfactants and polyphenols may improve the system's physical steadiness and dispersibility if polyphenols are introduced to the encapsulation system of ω‐3 PUFAs (Du et al., 2022).

## 4 UTILIZATION OF INNOVATIVE TECHNIQUES TO MITIGATE OXIDATION

The design of the interfacial structure is crucial in avoiding the oxidation of PUFAs. This is primarily accomplished by using chemical, enzymatic, and physical methods to alter interfacial layers made up of covalent and noncovalent compounds. Higher encapsulation efficiency for PUFAs was provided by Maillard reaction products (MRPs), which also reduced surface oil and improved PUFA oxidation protection in the microcapsules (Wang et al., 2020). It is common practice to generate polysaccharide polyphenol or protein–polyphenol couples via the free radical grafting process. Hydroxyl radicals are produced by redox processes and interact with certain residues on the chain of protein or polysaccharides to generate free radicals. Conjugates are produced as a result of a free radical and polyphenol reaction (Liu et al., 2016). The number of phenolic hydroxyl groups in conjugates is increased by the free radical grafting process, which also increases the conjugates' capacity to reduce free radicals and scavenge them (Gu et al., 2017). Intermolecular and intramolecular connections between biopolymers are encouraged by enzymatic crosslinking (Benjamin et al., 2014). It is a successful method for producing conjugates that might prevent the oxidation of lipid and increase PUFA stability in emulsions throughout storage. The microfluidic‐jet spray‐drying technique (MFJSD) has now been researched or established to manufacture microcapsules along with more pronounced particles and monodisperse particles to increase the uniformity of the drying of the ω‐3 fatty acid emulsions. Wang et al. (2017) produced WPI‐stabilized DHA oil microcapsules accompanied by monodisperse droplets via a 75 m microfluid aerosol nozzle using MFJSD technology (air flow rate 15 L/min, 160°C). The outcome shows that when the oil‐to‐WPI ratio is 1:2, the rate of encapsulation of DHA microcapsules manufactured by such method was 95%. During spray‐drying process, WPI could produce a crosslinking caused by heat network on the oil droplet surface, enhancing the protection of DHA. The electrospray technique, which is founded on the electrohydrodynamics of the dispersed materials and solutions, has been utilized to make ω‐3 PUFA microcapsules in low temperature in order to lessen the consequence of hot air flow on ω‐3 PUFAs. A range of antioxidant active ingredients are included in the combination obtained from both plants and animals, and they may work in concert to prevent the oxidation of ω‐3 PUFAs. A combination of flavonoids, tannins, and phlobatannins was isolated from carapace of coconut by Buamard and Benjakul (2017) using ethanol evaporation. The research found that when shrimp oil emulsion was stored at 30°C for 12 days, 200 mg/L extract from coconut shell greatly slowed down the deterioration of the lipids. Fascinatingly, the extract appeared to mix with SC, causing SC to become hydrophobic and redevelop antioxidants from hydrophobic ring, extending the antioxidant action of the phenolic compounds from the extract. Almost everything, the examined quality indicators, compositional characteristics, and kinetic framework of oxidative stability are impacted by refining. Analytical characteristics were impacted in varying degrees by the purifying stages of neutralization, bleaching, and deodorization. Our findings showed that the refining procedure led to considerable decreases in roughly 30% sterols, 86% primary oxidation products, 24% tocopherols, 96% free fatty acids, and 34% secondary oxidation products, among other substances. Bleaching enhanced the amount of stigmasta‐3,5‐diene, whereas neutralization removed FFAs, while deodorization increased the quantity of transfatty acids and the UV extinction coefficient K270 (Gharby et al., 2021).

## 5 ENHANCING SHELF LIFE AND OXIDATIVE STABILITY OF POLYUNSATURATED FATTY ACIDS WITH ANTIOXIDANTS

Fats and oils can be classified as PUFTs or monounsaturated. The World Health Organization and the Food and Agriculture Organization both agree, increasing the dietary intake of PUFAs lowers the chance of cardiovascular illnesses (Huang et al., 2016). Dietary sources richer in clinical and epidemiological studies, ω‐3 fatty acids have indeed been found to be beneficial research to provide significant health advantages. According to several research, the neuroprotective and anti‐inflammatory properties of ω‐3 fatty acids make them beneficial in the treatment of inflammation and autoimmune illnesses (Calder, 2001). Additionally, there is some proof that ω‐3 PUFAs are effective in treating schizophrenia and depression. ω‐3 fatty acids, including docosahexaenoic acid, are also essential for the growth of the human nervous system, the brain, and the eyes in fetuses and young children (Kolanowski et al., 2007). However, there is debate concerning the influence of ω‐6 fatty acids on health. For instance, despite linoleic acid's ability to decrease cholesterol, some scientists have hypothesized that linoleic acid consumption may increase the risk of heart attack (Lands, 2008). Additionally, it was found that giving linoleic acid to diabetic rats enhanced the sciatic nerve's nerve conduction velocity (Head et al., 2000). The primary diet of polyunsaturated is fish and vegetables. For instance, long‐chain PUFAs are abundant in fatty fish including tuna, trout, mackerel, and herring (Meyer et al., 2003). Mercury and other toxic metals put eating fish at risk. Additionally, these oils have a distinct fishy smell that lowers consumer acceptance and prevents them from being used in vegetarian and vegan diets (Timilsena et al., 2017). In addition to their fundamental nutritional value, they are used for a variety of other reasons, including their gastronomic, organoleptic, antimicrobial, anticancer, and antiaging effects (Bakry et al., 2016). When subjected to various environmental conditions, like humidity, oxygen, light, inorganic anions, and heat, PUFAs are vulnerable to isomerization, oxidation, polymerization, and the loss of volatile components due to their fragile chemical structure (Rustan & Drevon, 2001). In addition to producing hazardous chemicals, oxidative breakdown of the unsaturated fats during storage and processing can change important product qualities like flavor, color, nutritional value, and scent. The chain reaction process that causes the degradation of unsaturated fats essentially entails an induction stage; precisely, the interval of time before a sharp rise in the rate of oxidative stress is a gauge of oxidative stability and is known as the incubation time or induction period (Tan et al., 2002). The majority of highly corrosive oxidation products have undesirable side effects, including atherosclerosis, cancer, allergic reactions, and heart disease (Chen & Ahn, 1998; Kellens et al., 2007). In response to the decision from the American Red Cross, the Food and Drug Administration (FDA) has authorized irradiating red meat below low dosages to increase food safety business sources (Hollingsworth, 1998). Pork had previously been allowed to be irradiated (1 kGy) along with chicken (3 kGy) (Hampson et al., 1996) Thayer irradiation increases food safety; however, the approach will be approved by food processors based on how it affects other elements, such the Meat's oxidation and color stability. Fats begin to oxidize on their own as a result of irradiation, producing rancid off‐flavors. According to Ahn et al. (2000), the TBARS levels of pig patties that were packaged aerobically increased with increasing irradiation dose. According to Luchsinger et al. (1996), the TBARS readings for pork chops packaged aerobically rose with irradiation time and storage period. However, neither the irradiation dose nor the amount of time the pork chops were stored had an impact on the TBARS readings (Luchsinger et al., 1996). According to Stevenson (1994), food irradiation does not present any unique nutritional issues. Irradiation dosages up to 10 kGy do not appreciably impact the energy value of irradiation macronutrients in food. The color and oxidative stability of chilled (4°C) and minced beef were reported to be improved by a‐tocopherol acetate supplementation and vacuum packaging in prior work by Formanek et al. (1998). Modified atmospheric packaging (MAP) is the method of changing the initial gaseous environment that surrounds the food in a way that influences how the food and any food‐borne bacteria metabolize the food. The food itself, the gaseous atmosphere around the food, the packing materials, and external elements like temperature, handling environment, light, and infection may all affect the changes in the package atmosphere and the consequent product attributes, McMillin (2020). By raising the O_2_ level of MAP packages during storage, discoloration was increased. The aims of this study was to explore the color stability of irradiation minced beef and study the combination effect of vitamin E and antioxidant on irradiated beef. The variations in the composition of polyunsaturated fatty acids (PUFAs) reported as a share of the total concentration of chopped beef mince with storage duration and irradiation dose. Analytical techniques (Hunter Lab, alpha‐tocopherol content, pigment content, and TBARS of meat) were used as explained in earlier studies, unless otherwise noted (Formanek et al., 1998).

## 6 PREVENTION OF THE OXIDATIVE DEGRADATION OF POLYUNSATURATED FATTY ACIDS BY UTILIZING A SPHINGOID BASE

Better tactics have been proven for controlling lipid oxidation, particularly PUFA oxidation, including the use of antioxidants, inactivating pro‐oxidant metals, and limiting exposure to light and air. Micro‐ and nanoencapsulation have lately made significant advancements in the production of lipid emulsification and powders with enhanced oxidative stability (McClements & Decker, 2018; McClements & Jafari, 2018; Taneja & Singh, 2012). The increase in fish oil's oxidative stability has received a lot of attention due to the growing awareness in the health advantages of EPA and DHA, and technological advancements have made this possible and made it simpler to incorporate fish oil into supplements and nutritious foods (Bernardi et al., 2016; Suárez‐Jiménez et al., 2016; Walker et al., 2015). Even in the very early stages of oxidation, EPA and Vitamin d3 are extremely unstable and promptly breakdown to generate volatiles, which causes the creation of fishy and rotten aromas. Therefore, at the beginning of fish oil oxidation, an efficient strategy to scavenge the volatile chemicals may be required. This suggests that the Maillard response deserves a lot of research (Henna Lu et al., 2011). Major volatiles that cause fish oil to oxidatively degrade can be captured by the Maillard reaction of amine compounds like aldehydes, where the compound of the alkynes and the acid group can act as substrates. Sometimes, the products of the Maillard reaction exhibit antioxidant action and a synergistic interaction with tocopherols. As a result, amines could be employed as efficient agents to stop the oxidative degradation of EPA and DHA (Kazuo, 2019). Studies on PLs have been heavily influenced by the fact that amine compounds are powerful antioxidants for fatty acids. Shimajiri et al. (2013) contrasted the impact of various amine compounds, such as sphingolipids (SLs), on fatty acid TAG oxidation and a sphingoid base (SPG) as their backbone. According to a recent study, adding SPG or ‐tocopherol alone did not entirely prevent the formation of volatile chemicals such as propanal and acrolein during the 380‐h incubation of purified fatty acid TAG at 50°C. Instead, volatiles immediately surged at the early stage of oxidation, Uemura et al. (2016). According to several model investigations, SPG and aldehydes can react quickly to produce antioxidative Maillard reaction products reported by Uemura et al. (2016). While nonoxidized tricaprylin, which included the same amount of α‐tocopherol and SPG as that found in the fatty acid TAG, contained the same quantity of α‐tocopherol and SPG as that found in the fatty acid TAG, such antioxidants were not present. Therefore, only SPG was unable to create any antioxidants to prevent the oxidation of fish oil TAG. Any oxidation products, like aldehydes, are required for the synthesis of antioxidant activities Maillard reaction compounds. Acrolein is identified as a prominent oxidizing agent at the preliminary phase of fish oil TAG oxidation (Shibata et al., 2015).

## 7 NANOENCAPSULATION

### 7.1 Nanoparticles

Nanoparticles are broadly defined as materials with at least one dimension <100 nm; these substances can be 0D, 1D, 2D, or 3D based on their overall geometry (Khan et al., 2017). Due to the use of natural ingredients with a three‐dimensional structure in the food industry, the carriers are practically three‐dimensional. Dietary components called ω‐3 polyunsaturated fatty acids play a role in the mitigation of neoplastic, inflammatory, and cardiovascular disorders. ω‐3 PUFA‐containing nanoformulations have recently been developed using a multidisciplinary technique, depending on the latest observations in biotechnology, lipid biochemistry, nutritional science, and the biology of cancer and inflammation (Islam, Saeed, Afzaal, & Ahmad et al., 2022). The goal of these preparations is to preserve these fatty acids from deterioration, boost their bioactivity and distribution to target cells, and, consequently, improve their bioactivity. In certain instances, these nanoformulations were created to deliver ω‐3 PUFAs together with various nutraceuticals or traditional/innovative medications reported by Serini et al. (2019).

### 7.2 Lipid nanoparticles

Lipid nanoparticles make excellent transport and protection vehicles for hydrophobic bioactives. They are highly capable of producing on a vast scale. Numerous lipid nanoparticles have been proposed, including lipid nanocapsules, solid lipid nanoparticles (SLN), nanoemulsions, nanoliposomes, and nanostructured lipid carriers (NLC) (LNC). Investigations were done into how the choice of surfactant affected the solid–lipid–nanoparticle emulsions that included encapsulated beta‐physical carotene's and chemically sustainability. By homogenizing 10% w/w lipid phase (1 mg/g β‐carotene in transporter lipid) but also 90 percent w/w aqueous (cosurfactant + surfactant) at 75°C and pH 7 and then chilling to 20°C, oil‐in‐water suspensions were created. Utilizing aqueous phase including several surfactants, which are water‐soluble, including Tween 80, 2.4% w/w or 1.4% w/w, Tween 60 high‐melting lecithin, cosurfactant (0.6% taurodeoxycholate), and 2.4% w/w low‐melting lecithin, it was determined how the kind of surfactant affected the results. Utilizing whether a lipid with a high‐melting point (tripalmitin), which produces solid particles, or even a lipid with a lower melting point, which generates liquid droplets, the effects of the transporter lipid's physical configuration were examined. Solid particles made with high‐melting surfactants (Tween 60 and HM lecithin) were shown to have more α‐crystals than those made with low‐melting surfactants (Tween 80 and LM‐lecithin). The suspended particles encapsulated with HM lecithin were the only exception to the treatments' resistance to particle agglomeration after storage for 21 days. These findings imply that (1) HM lecithin or LM when using liquid transporter lipids and (2) HM lecithin when using solid transport lipids may stabilize β‐carotene reported by Helgason et al. (2009).

### 7.3 Nanoemulsions

Multiphase colloidal dispersions known as nanoemulsions are created as nanoscale droplets by physically or chemically rupturing one liquid into another immiscible liquid. Liquid lipids make up nanoemulsions. Nanoemulsions, which have diameters smaller than visible wavelengths, are optically transparent in contrast to microemulsions, which scatter visible light many times and have an opaque white appearance (Vickers, 2017). The creation of functional foods has undergone a revolution thanks to nanoemulsions, which have opened up new ideas for nutrient encapsulation (Kumar & Sarkar, 2018). In addition to their nutritional value, food products also offer health‐promoting or preventative qualities. According to research, the synergy or connections of biologically active compounds and other essential nutrients present in food products account for the health‐promoting capacity of food stuff (Alshahrani, 2022). Furthermore, these bioactive chemicals might not be sufficient to maintain their effects due to their limited availability and long‐term sustainability. As a result, recent times have seen a lot of interest in the use of nanotechnology in culinary applications (Islam, Saeed, Afzaal, & Hussain et al., 2022).

### 7.4 Lipid nanocapsules

Lipid nanocapsules are a new class of carriers that combine the structures of polymeric nanocapsules with liposomes (LNCs). Contrary to liposomes, which can occasionally be produced using organic solvents and are unstable in body fluid and leaky, LNCs are prepared using solvent‐free and soft‐energy techniques, and they have excellent stability. Because of its tiny size (between 25 and 100 nm) and capacity to encapsulate both hydrophobic and lipophilic bioactive substances, this nanocarrier is made from generally regarded as safe (GRAS) materials and is a great substitute for emulsions, microemulsions, or liposomes (Martins et al., 2012).

## 8 MICROENCAPSULATION

Polyunsaturated fatty acids; DE, dextrose equivalent There is a contemporary trend towards living a healthy lifestyle, which includes increased consumer knowledge of what individuals eat and the benefits of particular foods in maintaining their health. Dietary changes can help you stay healthy by an exceptional chance to innovate so‐called functional food (Sheehy & Morrissey, 1998). These products frequently confront the food industry by engineering new challenges. It is necessary to incorporate both new and existing substances into food systems because they progressively deteriorate there and lose their functionality or turn dangerous due to oxidation reactions. Ingredients and components may also interact with the presence in the food supply chain, which may restrict bioavailability or alter a product's color or flavor. In numerous circumstances, microencapsulation can overcome these tasks. Gases, liquid droplets, or tiny solid particles are encased in a covering by a process known as microencapsulation. Polymers are frequently used as the matrix or shell materials. Microencapsulation technologies are classified into two types: those that spray a liquid phase into a gas suspension medium (spray‐drying or spray‐cooling) and those that use a liquid suspension medium (complex coacervation, interface, and in situ polymerization, or solvent evaporation from emulsions), coextrusion, or fluidized‐bed coating. Excellent descriptions of the various methods for creating microcapsules may be found in several publications (Shahidi & Han, 1993). Pharmaceuticals, pesticides, scented strips, carbonless copy paper, and other products all use microcapsules (Afzaal, 2023). Growing interest is being shown in the use of microencapsulation in the food business to safeguard, isolate, or regulate the release of a specific ingredient. A liquid can be changed into a powder to use different substances. The encapsulating of flavors is one of the most common food applications. Shahidi and Han (1993) provide a nice overall summary of components in encapsulated foods. Vitamin C, which is frequently added to a range of food products as an antioxidant or a vitamin supplement, is a suitable example (Kirby et al., 1991). Foods frequently include ascorbic acid as an antioxidant. It could be utilized to protect emulsion‐type meals by being combined with vitamin E in a liposome, which can have a potent antioxidant effect (Reineccius, 1995). The liposome wall incorporates vitamin E (Berrocal & Abeger, 1999).

## 9 HEALTH BENEFITS AND FUTURE PERSPECTIVE

To improve the utilization of natural resources, changes must be made to the structure and administration of our food systems (Maqbool et al., 2023). A growing demand for food items from pasture‐based production systems is a reflection of consumers' growing concern for the environment, animal welfare, and the source and manner of food production. The most cost‐effective method of feeding milk cows is grazed grass. Milk products are a significant source of fatty acids (FAs) in the human diet; hence, the composition of milk's fatty acids (FA) is of great importance. Farm milk composition is crucial since the FA profile of dairy fats is similar to that of the animal fats from where these products are derived (Dhiman et al., 1999). Cow milk's fat content, FA composition, and concentration are mostly influenced by the diet of the cows (Kalač & Samková, 2010). Numerous reviews have been written about the FA profiles of milk fat, including Chilliard et al. (2007). It has frequently been discovered that milk from ruminants that graze has greater levels of polyunsaturated acids (PUFAs) compared with ruminants maintained inside systems of production based on grazing. However, biological systems (Schwendel et al., 2015) do not have unique says that milk has more PUFA. Supplemental feeds such as oilseeds, fish, or algae sources can be used to boost these components in confined feeding, although doing so may have negative impacts on the composition of unsaturated fatty acids (UFAs) (Aldai et al., 2013). Polyunsaturated fatty acids, sometimes known as “PUFAs,” are both necessary and nonessential parts of cell membrane phospholipids and act as precursors to eicosanoids, a class of hormone‐like inflammatory mediators, Table 1. Recent studies have shown that long‐chain PUFAs can modulate a variety of transcription factors to control cellular metabolism at the nucleus level. It is believed that this effect on gene regulation contributes to the metabolic connection between diet PUFA intake, wellbeing, and the development of chronic illnesses. Additionally, EPA and DHA have positive effects on insulin resistance by increasing the release of the anti‐inflammatory adipokine adiponectin. In conclusion, n‐3 PUFAs have a variety of health advantages that are at least partially mediated by their anti‐inflammatory effects; as a result, their consumption, particularly from dietary components, should be promoted. Because nutritional wellbeing is a continuous force for growth and wellbeing, as well as the optimization of genetic potential, it is important to maintain the nutritional quality of food. Therefore, dietary quality should be taken into account to address the issues of widespread food insecurity. Millets were discovered to have a high nutritional content that is comparable to that of main grains like wheat and rice, in addition to their cultivation advantages (Parameswaran & Sadasivam, 1994).

**TABLE 1:** Health benefits of PUFA.

PUFA	Health benefits	Reference
ω‐3 fatty acids		
α‐linolenic acid (ALA)	Promotes cardiovascular functioning, blood pressure regulation, and neurological problems; anti‐inflammatory and antiarrhythmic characteristics	Blondeau et al. (2015) and Gogna et al. (2023)
Eicosapentaenoic acid (EPA)	Anti‐inflammatory qualities, fewer serious coronary incidents, and better antiplatelet actions increase cardiovascular performance. Protects against severe brain damage	Narayan et al. (2006) and Swanson et al. (2012)
Docosahexaenoic acid (DHA)		
ω‐6 fatty acids		
Linoleic acid (LA)	Decrease body fat with increasing lean body mass, and modify immunological and/or inflammatory reactions to decrease the formation of atherosclerosis	Kim et al. (2016)
Arachidonic acid (AA)	The neurological system (cognitive and brain processes), skeletal muscle, and immune processes all require it for proper growth and functioning	Tallima and El Ridi (2017)

## 10 CONCLUSION

Polyunsaturated fatty acids may be derived from a variety of sources and should be included in the daily diet for wellbeing and the protection of many illnesses. Globally, ω‐6 and ω‐3 PUFAs are the most often taken supplementation in the type of fatty acid‐rich foods. Nutrition has a significant impact on the metabolism and physiological performance of the organism. The ω‐3 and ω‐6 PUFAs found in both terrestrial and marine environments have received special attention. LA and ALA are essential fatty acids that stimulate the production of AA, EPA, and DHA in the human body, all of which play critical roles in maintaining physiological homeostasis. Eicosanoids, which are bioactive signaling lipids, also regulate homeostatic processes.

## CONFLICT OF INTEREST STATEMENT

The authors declare no conflict of interest.

## ETHICS STATEMENT

This article does not involve humans or animals.

## CONSENT TO PARTICIPATE

All the co‐authors are willing to participate in this manuscript.

## CONSENT FOR PUBLICATION

All authors are willing for the publication of this manuscript.

## Data Availability

Even though adequate data have been given, all authors declare that if more data were required, then the data will be provided on a request basis.
